# Supplementary material for: Providing person-centered care for patients with complex healthcare needs: A qualitative study
Source: PLoS One. 2020 Nov 16;15(11):e0242418. doi: 10.1371/journal.pone.0242418 (PMC7668580; doi:10.1371/journal.pone.0242418)

**Modular service architecture:
Case B**

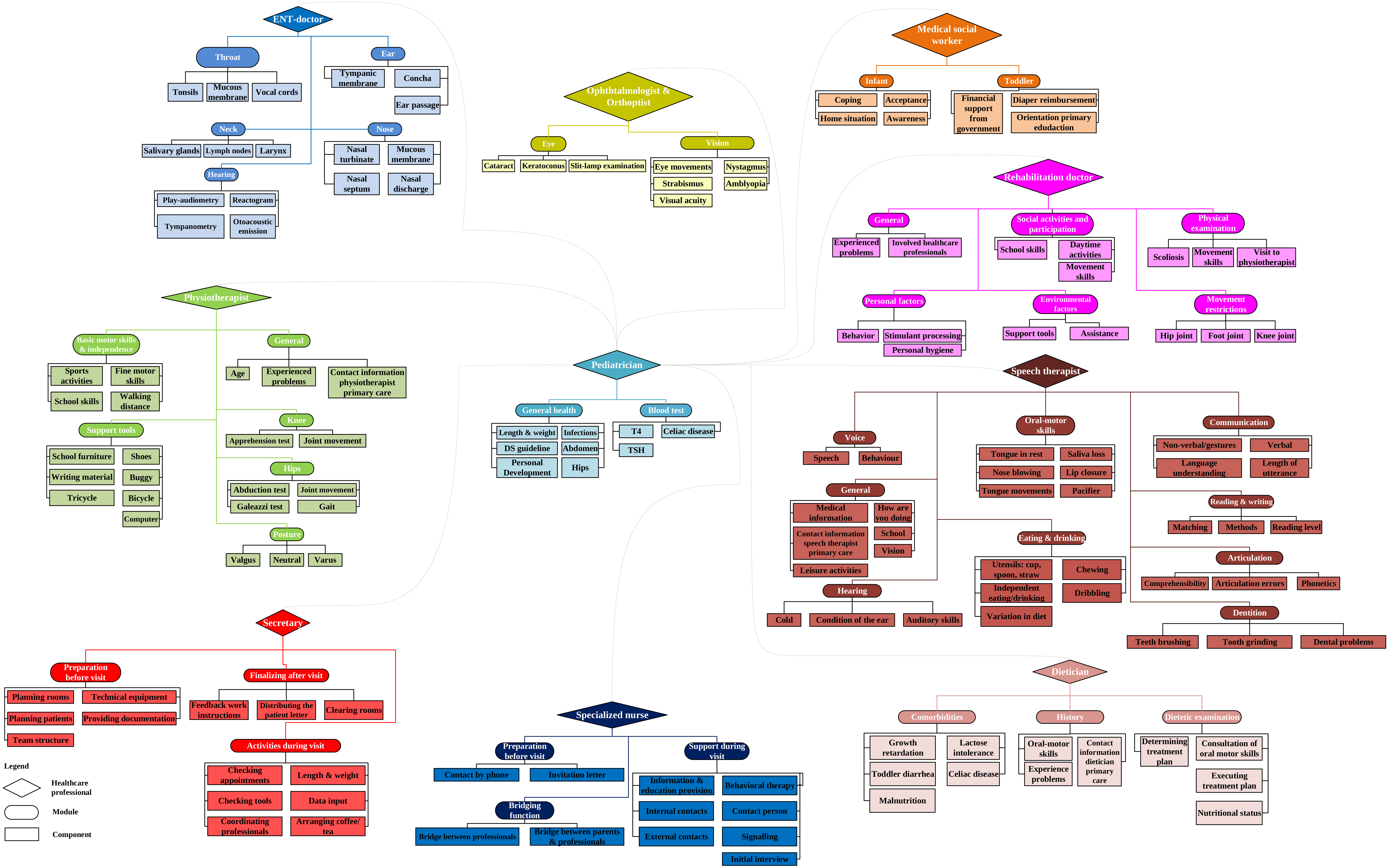

# Modular service architecture: Case C

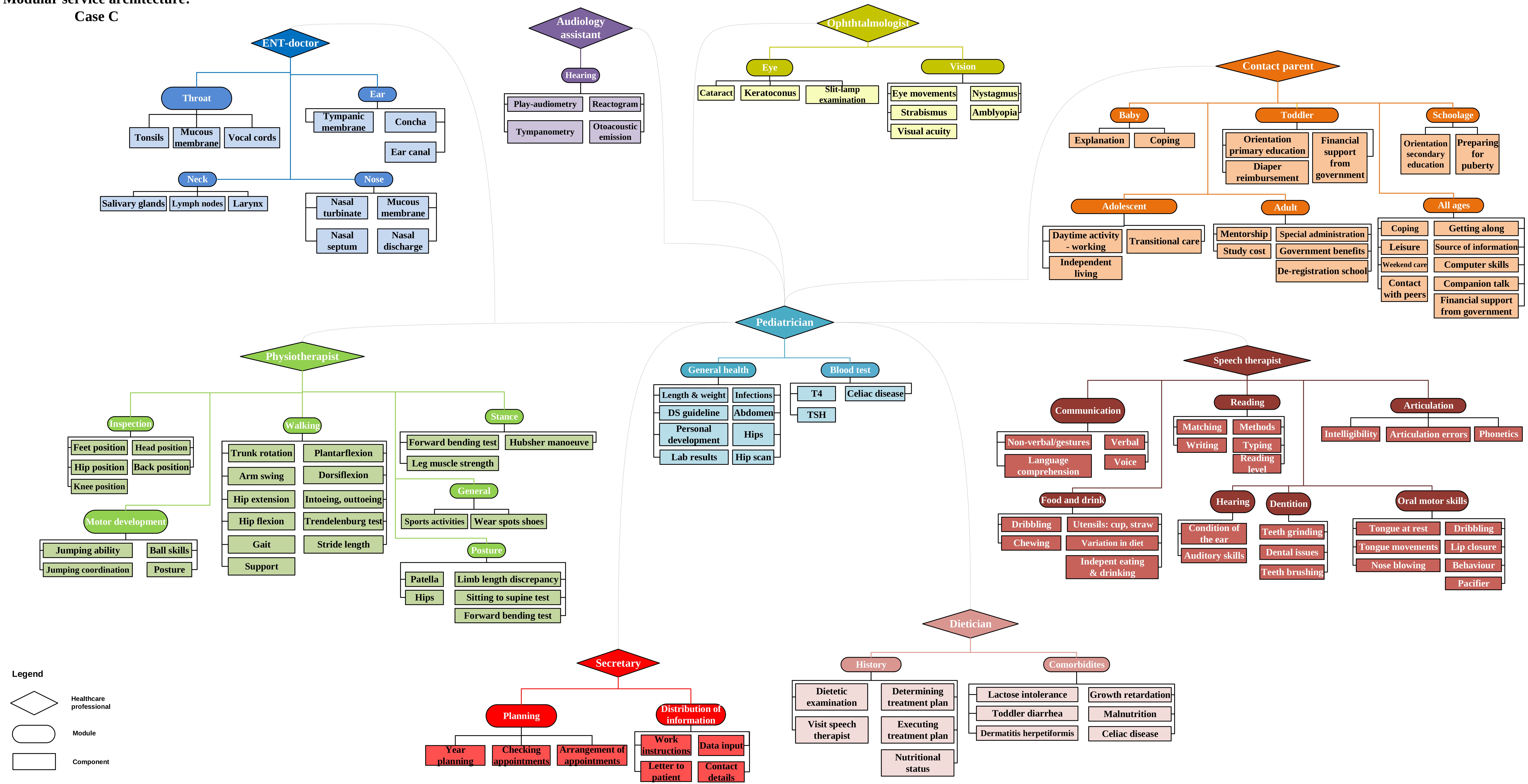

# Modular service architecture: Case D

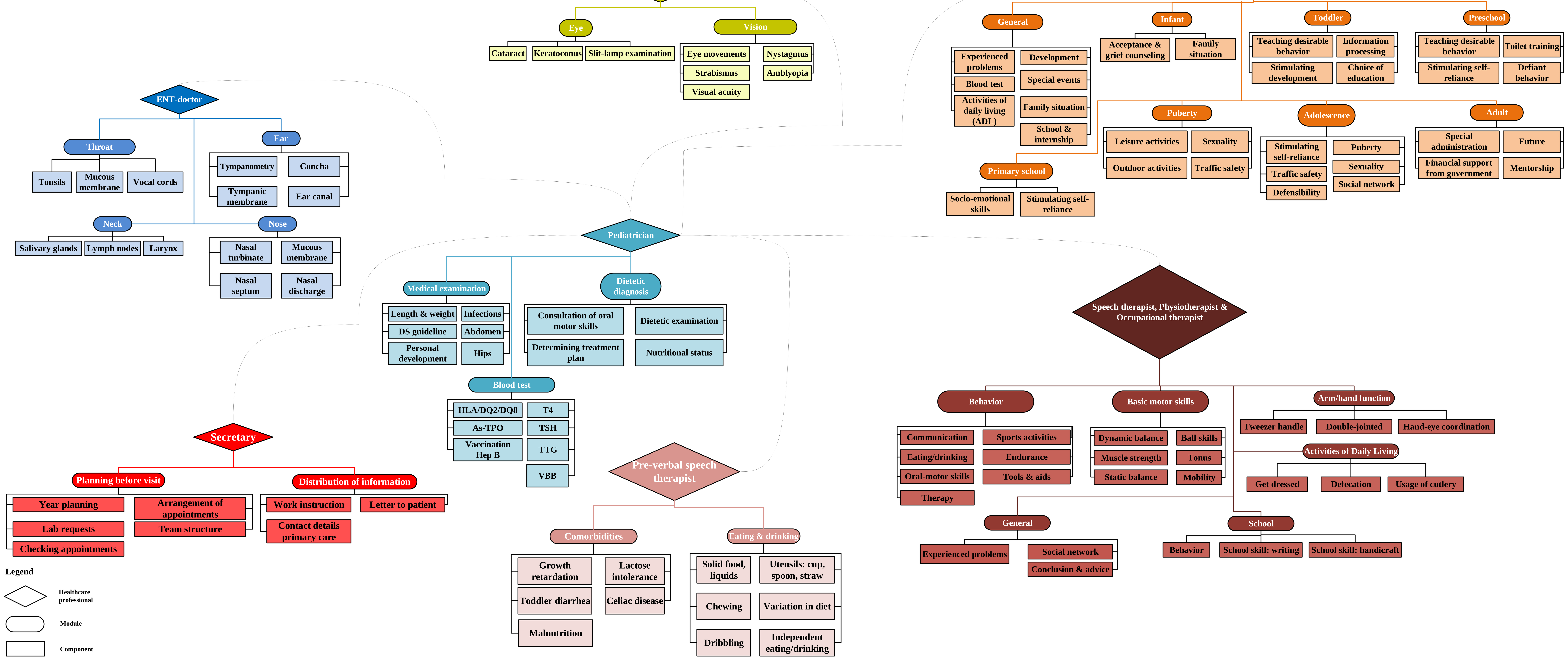

Supplement: S1 Fig — (PDF) [file pone.0242418.s001.pdf]
