# Supplementary material for: Providing person-centered care for patients with complex healthcare needs: A qualitative study
Source: PLoS One. 2020 Nov 16;15(11):e0242418. doi: 10.1371/journal.pone.0242418 (PMC7668580; doi:10.1371/journal.pone.0242418)
Supplement: S2 File — (DOCX) [file pone.0242418.s003.docx]

**S2 File. Topic list.**

1. General questions

- 1.1 What is your name and age?
- 1.2 Can you briefly describe your current job and your role in the Downteam?

2. Modular organization

- 2.1 Can you explain how the Downteam works?
  - What (care) components does your discipline comprise?
  - What parts of the care that you provide can also be provided by other specialists in the Downteam?
  - What do you think of the current multidisciplinary organization of your Downteam?
- 2.2 What processes have been more or less standardized in the care for people with DS?
- 2.3 What components of your role would you describe as being more standardized, and which ones would you describe as more tailored to the patient?
  - In which way do you attempt to provide patient-centered care?
- 2.4 Which healthcare professionals are not part of the Downteam, but are connected to the Downteam?
- 2.5 Which important information transfers take place within the Downteam and outside it? How do these take place? Can you give some examples?
  - How does the information transfer from the Downteam to primary care take place?
  - How does the information transfer from the Downteam to a care organization take place?
- 2.6 How is the electronic health record (EHR) employed? Who has access to the EHR? What communication outside of the EHR is important to ensure a good provision of care?

3. Information exchange

- 3.1 How would you describe the information exchange within the Downteam? By this I mean whether the information is complete, accurate and up to date.
- 3.2 How would you describe the information exchange between the Downteam and primary care organizations? By this I mean whether the information is complete, accurate and up to date.
- 3.3. Whom or what has been designated as responsible for the coordination of the care for people with DS? If so, how did this happen?
- 3.4 Would the designation of a single organization or person to bear responsibility for the coordination of care for people with DS influence the coordination of activities?
- 3.5 How do you experience the contact with other caregivers/specialists within the Downteam?
  - How do you experience the contact with other specialists outside of the Downteam?
  - How do you experience the contact with primary care?
- 3.6 How are work processes and activities coordinated between your specialization and primary care?
- 3.7 On a scale from 1 to 10, how would you rate the degree of coordination in the care for people with DS at this moment?
  - What are your reasons for this rating?
- 3.8 Do you see any room for improvement with regard to the coordination of activities between the Downteam and primary care?
- 3.9 Who schedules the appointments/examinations for patients? Who checks whether these have indeed taken place?

4. Patient centeredness

- 4.1 Are patient discussions or progress discussions held outside of the multidisciplinary consultation, in which the patient’s care plan is discussed?
- 4.2 To what extent is the care tailored to the specific needs of patients (e.g. age, personal preferences etc.)?
- 4.3 Who is responsible for communication with and information provision to the patient?
- 4.4. To what extent do you consult the information in the patient’s medical history?
- 4.5 Please elaborate on the following statement: The patient and/or the patient’s parents are capable of taking on a coordinating role
- Which things that patients have a need for are given insufficient attention?
  - How do patients get the opportunity to express their needs, wishes or demands when they visit the Downteam?
  - Do you see possibilities for (more) patient participation? Why/why not?
