# Supplementary material for: Providing person-centered care for patients with complex healthcare needs: A qualitative study
Source: PLoS One. 2020 Nov 16;15(11):e0242418. doi: 10.1371/journal.pone.0242418 (PMC7668580; doi:10.1371/journal.pone.0242418)
Supplement: Attachment — Submitted filename: Response to Reviewers.docx [file pone.0242418.s005.docx]

Reviewer: 2

**Q1: Many thanks for addressing all my comments from the previous round. I would suggest that the authors address the following comments in their next round of revisions.**

R: We are happy to hear that we addressed the comments in an appropriate manner. We will go over the remaining comments to clarify the issues point by point below.

**Q2: Please proof-read the whole manuscript to address typos and spelling mistakes**

R: We have addressed an experienced copy editor to proofread the whole manuscript. The copy editor is a native English speaker and she has addressed the typos and spelling mistakes. Please see the manuscript accordingly.

**Q3: Please ensure that all references are complete and formatted consistently**

R: We have ensured that all references are complete and formatted consistently. Please see the reference list for our changes.

**Q4: Please discuss in more detail how your findings may be applied to other types of (healthcare) services to offer some degree of generalisability and links to possible future research**

R: We incorporated more detail about how our findings can be applied in other types of (healthcare services) to offer some degree of generalizability and links to possible future research. We now provide two examples of other types of services: legal services and higher educational services.

*C: We also believe that the MSA approach is applicable in many other types of complex services such as legal services or higher educational services. For example, when clients face a legal conflict (divorce, termination of employment etc.) they can make use of a variety of providers in dealing with their conflict. Each provider is responsible for providing a subset of services for the client and collectively the providers offer the service that fits with the client’s needs and wishes. Approaching legal services from a modular perspective allows for the decomposition into components and modules, resulting in transparency on the supply side of legal services. The modular perspective is also relevant for clients because although accurate information about legal services becomes increasingly available online [52], the clients are not fully aware of what each legal provider can offer them. This results in legal services that are not completely tuned to the needs and requirements of clients. In higher educational services in many countries there has been an increasing focus on individualized instructions, despite the increasing number of students [53]. As a result, there is a need to make higher education services available to large number of students and, at the same time, offer an individualized learning package for each student. A modular approach could help in dealing with this issue, as it provides opportunities to offer curricula or interdisciplinary programs that are designed based on modules, where each student’s program is tailored to their individual needs and wishes [54]. Future research should test whether the MSA approach is feasible in these complex service settings.* (Discussion, page 23-24)

Additional reference:

52. Giannakis M, Doran D, Mee D, Papadopoulos T, Dubey, R. The design and delivery of modular legal services: Implications for supply chain strategy. International Journal of Production Research. 2018;56(20): 6607-6627. doi:10.1080/00207543.2018.1449976

53. Goldschmid B, Goldschmid ML. Modular instruction in higher education: A review. Higher Education. 1973;2: 15-32. doi:10.1007/BF00162534

54. Turnbull W, Burton D, Mullins P. Strategic repositioning of institutional frameworks’: Balancing competing demands within the modular UK higher education environment. Quality in Higher Education. 2008;14(1): 15-28. doi:10.1080/13538320802011474

- A marked-up copy of your manuscript that highlights changes made to the original version. You should upload this as a separate file labeled 'Revised Manuscript with Track Changes'.
- An unmarked version of your revised paper without tracked changes. You should upload this as a separate file labeled 'Manuscript'.

If you would like to make changes to your financial disclosure, please include your updated statement in your cover letter. Guidelines for resubmitting your figure files are available below the reviewer comments at the end of this letter.

If applicable, we recommend that you deposit your laboratory protocols in protocols.io to enhance the reproducibility of your results. Protocols.io assigns your protocol its own identifier (DOI) so that it can be cited independently in the future. For instructions see: <http://journals.plos.org/plosone/s/submission-guidelines#loc-laboratory-protocols>

We look forward to receiving your revised manuscript.

Kind regards,

Janhavi Ajit Vaingankar
Academic Editor
PLOS ONE

Journal Requirements:

When submitting your revision, we need you to address these additional requirements.

1. Please ensure that your manuscript meets PLOS ONE's style requirements, including those for file naming. The PLOS ONE style templates can be found at

https://journals.plos.org/plosone/s/file?id=wjVg/PLOSOne_formatting_sample_main_body.pdf and

https://journals.plos.org/plosone/s/file?id=ba62/PLOSOne_formatting_sample_title_authors_affiliations.pdf

2. Please provide the dates of participant recruitment in your manuscript.

3. We note that you have indicated that data from this study are available upon request. PLOS only allows data to be available upon request if there are legal or ethical restrictions on sharing data publicly. For information on unacceptable data access restrictions, please see http://journals.plos.org/plosone/s/data-availability#loc-unacceptable-data-access-restrictions.

In your revised cover letter, please address the following prompts:

a) If there are ethical or legal restrictions on sharing a de-identified data set, please explain them in detail (e.g., data contain potentially identifying or sensitive patient information) and who has imposed them (e.g., an ethics committee). Please also provide contact information for a data access committee, ethics committee, or other institutional body to which data requests may be sent.

b) If there are no restrictions, please upload the minimal anonymized data set necessary to replicate your study findings as either Supporting Information files or to a stable, public repository and provide us with the relevant URLs, DOIs, or accession numbers. Please see http://www.bmj.com/content/340/bmj.c181.long for guidelines on how to de-identify and prepare clinical data for publication. For a list of acceptable repositories, please see http://journals.plos.org/plosone/s/data-availability#loc-recommended-repositories.

We will update your Data Availability statement on your behalf to reflect the information you provide.

4. Please amend your manuscript to include your abstract after the title page.

[Note: HTML markup is below. Please do not edit.]

Reviewers' comments:

Reviewer's Responses to Questions

**Comments to the Author**

1. Is the manuscript technically sound, and do the data support the conclusions?

The manuscript must describe a technically sound piece of scientific research with data that supports the conclusions. Experiments must have been conducted rigorously, with appropriate controls, replication, and sample sizes. The conclusions must be drawn appropriately based on the data presented.

Reviewer #1: Yes

Reviewer #2: Partly

2. Has the statistical analysis been performed appropriately and rigorously?

Reviewer #1: N/A

Reviewer #2: N/A

3. Have the authors made all data underlying the findings in their manuscript fully available?

The [PLOS Data policy](http://www.plosone.org/static/policies.action#sharing) requires authors to make all data underlying the findings described in their manuscript fully available without restriction, with rare exception (please refer to the Data Availability Statement in the manuscript PDF file). The data should be provided as part of the manuscript or its supporting information, or deposited to a public repository. For example, in addition to summary statistics, the data points behind means, medians and variance measures should be available. If there are restrictions on publicly sharing data—e.g. participant privacy or use of data from a third party—those must be specified.

Reviewer #1: Yes

Reviewer #2: Yes

4. Is the manuscript presented in an intelligible fashion and written in standard English?

PLOS ONE does not copyedit accepted manuscripts, so the language in submitted articles must be clear, correct, and unambiguous. Any typographical or grammatical errors should be corrected at revision, so please note any specific errors here.

Reviewer #1: Yes

Reviewer #2: Yes

5. Review Comments to the Author

Please use the space provided to explain your answers to the questions above. You may also include additional comments for the author, including concerns about dual publication, research ethics, or publication ethics. (Please upload your review as an attachment if it exceeds 20,000 characters)

Reviewer #1: Overall
Overall this is a well written paper reporting original and important results, highly suitable for publication with some revision as indicated. The study design is appropriate and well described. Adherence to and inclusion of the COREQ is a strength. More could be explored in the Discussion section about of the differences between health professional and parent respondents, and applicability or otherwise of results to other settings.
Background
The concept and the term ‘modular service architecture’ is unlikely to be familiar to most readers of the journal. Add in more detail here about the principles that underpin MSA and justify more fully why and how these principles are applicable to health care for those with complex conditions.
More detail is also required about DS as a complex condition in the introduction. Although a complex condition with heterogeneous manifestations, nevertheless the constellation and combination of functional problems are reasonably predictable for this group. See comment about following this up under Discussion below.
Methods
How and why were the four Downteams selected as cases? Are they typical/atypical of all such teams? What diversity do they display?
Over what time period were all the interviews undertaken? The first 65 (reaching data saturation) and then the remainder? Were there any significant changes in the health system or staffing during the data collection period?
Provide more detail about how the collection and analysis of the direct observational data was undertaken. How did this feed into the within-case analysis?
Results
The figures provided are excellent, if complex. High quality reproduction of these is going to be crucial to readability – this has been mentioned in comments to the editor.
It would be worth pointing out in a little more detail in the text where the most striking gaps and duplication in care have been exposed by the research.
With regard to quotes – excellent to see the different health professional (HP) respondents identified by discipline but please also assign a unique identifier to each respondent. e.g. dietitian A; physiotherapist B – so we as readers know it is not merely the same dietitian being quoted throughout. And same again, with each Parent respondent – Parent a, Parent b, and so on.
Were there clear differences between the medical respondents and other HP respondents? Was there variation within these sub-groups? Clarify in the text.
Discussion
Referring to the point about DS as a particular sort of complex condition, compare this briefly to the patients with widely variable multi-morbidity, where there is not so often a common origin for the constellation of problems, and functional issues will be far more widely diverse. MSA approaches are likely more difficult to apply in such settings, so expand your explanation in the limitations paragraphs to cover this more clearly. Perhaps such an approach is only feasible for a particular, well-defined complex condition? Or not?
One of your key findings is around the stark difference between the patient and health professional perspectives. This deserves more prominence in the Discussion, since your main audience are health professionals who may find this challenging. You could refer more widely to other studies in different contexts – e.g.
Young, J., Poole, U., Mohamed, F., Jian, S., Williamson, M., Ross, J., Jaye, C., Radue, P., & Egan, T. (2019). Exploring the value of social network 'care maps' in the provision of long-term conditions care. Chronic Illness. Advance online publication. doi: 10.1177/1742395319836463
Abstract
Generally clear and an accurate representation of the paper, but add in a few more words about what MSA is, in line with the request about this in the Background section above.
Style/writing/references
In the main, the paper is structurally sound and reads well. Check again throughout for word choice and grammatically correct English. I have indicated below some of the more obvious corrections needed:
Line 51 – ‘Service modularity involves the decomposition of a complex service into modules…’ – the word ‘decomposition’ is probably better replaced with ‘deconstruction’, but check meaning and English language for clarity
Line 91 – ‘These four Downteams are well known in the field and provide a good of all Downteams in the Netherlands,…’ - correct the sentence
Line 109 – ‘The data were collected by VP…’ – explain who VP is – e.g. ‘author VP’ or ‘researcher VP’
Line 141 – ‘Three researched discussed and assessed the…’ – correct the sentence
Line 272-3 – ‘The modular perspective enabled us to fully decompose the healthcare provision into modules…’ – correct the word ‘decompose’ (similar problem to Line 51 comment above)

Reviewer #2: The paper started to address an interesting and timely research area, focusing on providing person-centered care for patients with complex healthcare needs. The authors draw on some prior studies, but do not offer a very critical literature analysis. This is needed to strengthen the paper’s argument and draw out the gaps they seek to address. Also, the paper needs to present a much deeper methods and findings sections as well as stronger discussion and conclusion sections in order to offer value to the reader. Overall, the manuscript makes some very interesting points and I realise that a lot of work went into this study. Nevertheless, I see room for improvement which will help to enhance clarity, readability, practical and theoretical contributions. The following paragraphs address each section of the paper in more detail and provide suggestions on how to revise the paper.

Major concerns:

Introduction:
While the author(s) establish some links to some extant literature, author(s) need to establish a more coherent framework for the overall paper. That means, the introduction should clearly indicate the need for this paper in relation to extant research studies. Which gap(s) are authors trying to address here? In order to link back to relevant extant literature, work on services, service complexity (Kreye et al. and work by Andy Neely et al.) and customization/modularization needs to be further emphasized (which gaps you seek to address and what you found). This would help to further ground your study in extant work and help you to motivate your study further.

Conceptual background & Theoretical development
The authors need to establish some clearer links to extant service literature (e.g. please see suggested key references). The author(s) should clearly draw out what is known for their key concepts. For instance, who is responsible for breaking up modules and components in your setting?; How easily can this be done?; How would this translate to other setting and what does prior work say about all this? This section needs much more information and guidance for reader to position the gaps in prior work. Also, given the nature of the services you are investigating, the authors should at least acknowledge prior work on knowledge intensive professional (KIP) services and their unique characteristics and features.

Methods and Analysis:
Overall, this section started to offer some insights, but I see room for improvements to obtain robust results and strengthen the paper’s argument. The authors should address the following concerns:

\# Please clearly tell the reader about your sampling logic with regards to interviewees, context/sector and type of services.
\# here you need to also bring out the differences between interviewees/observations etc. At the moment, most of the data converge and no real differences are noticeable.
\# Clearly and in more detail explain your data analysis steps (what have you analysed first and how)
\# A lot of the results section “tells” reader about your data rather than “shows” your data. Here, some data tables/figures and much more of a narrative would help to convince the reader that your findings support your conclusions.
\# What is the impact of different skills/capabilities of professionals when talking about/judging modules and components?
\# Parents may struggle to judge (as we are talking about KIP services) the value of medical services. How can you really compare data from healthcare professionals and non-professionals? Wouldn’t you expect differences in perception and value?
\# How have you measured the value (e.g. information exchange) of new/revised MSAs?

Discussions and Conclusions:
Derived from a conceptual background section which did not clearly draw out the gaps the paper seeks to address, the discussion and conclusion sections do offer only some additional value to the reader as it stands. The authors need to offer more fine-grained results here and discuss what they intended to find out in the introduction section (link to research question; overall aim of the paper). The authors should also link their findings to prior studies. Overall, the authors need to draw out clearly what the theoretical contributions are and how they add to the existing body of knowledge. This section also needs to clear link back to extant studies to offer some clear value to the reader. This should then help to derive some more insightful practice and policy implications.


Useful references:
Kreye et al. (2015). Servitizing manufacturers: The importance of service complexity and contractual and relational capabilities. Production Planning & Control, Vol. 26 No. 14-15, pp. 1233-1246.

Neely, A., D. McFarlane, and I. Visnjic 2011. “Complex Service Systems – Identifying Drivers, Characteristics and Success Factors.” 18th European Operations Management Association Conference, Cambridge, UK, July.

6. PLOS authors have the option to publish the peer review history of their article ([what does this mean?](https://journals.plos.org/plosone/s/editorial-and-peer-review-process#loc-peer-review-history)). If published, this will include your full peer review and any attached files.


If you choose “no”, your identity will remain anonymous but your review may still be made public.


**Do you want your identity to be public for this peer review?** For information about this choice, including consent withdrawal, please see our [Privacy Policy](https://www.plos.org/privacy-policy).

Reviewer #1: **Yes:** Professor Sue Pullon

Reviewer #2: No
